# Exploiting the bootstrap method to analyze patterns of gene expression

**DOI:** 10.1186/1471-2105-15-S10-P19

**Published:** 2014-09-29

**Authors:** Nam S Vo, Vinhthuy Phan

**Affiliations:** 1 Department of Computer Science, University of Memphis, Memphis, TN 38152, USA

## Background

High-throughput technologies like microarrays or the recent RNA-Seq provide large amounts of data for gene expression studies. Although there have been diverse methods to design gene-expression experiments and analyze gene-expression data, the prediction of true patterns of gene expression in case of having few samples remains a challenging problem [1,2].

## Materials and methods

We propose a method to predict response patterns of gene expression studies in the case of small sample size using a bootstrap method [3]. Our approach adopts partially order sets (*posets*) to represent gene patterns, which are determined based on pairwise comparisons [4].

## Results

We show that patterns that are *not linearly orderable* cannot be true patterns of gene response to treatments. From this result, we propose a strategy using bootstrap resampling to infer true responses of non-linearly-orderable patterns. Our experiments showed that this method produced gene lists with more biological functional enrichment than those obtained without bootstrap resampling.

## Conclusions

Our method is useful in designing cost-effective experiments with small sample sizes. Researchers can still use a small sample size to determine true patterns for most genes. For highly-variantly expressed genes, their true patterns can be identified using the proposed method.
